# ABCG2 Expression as a Potential Survival Predictor in Human Gliomas

**DOI:** 10.3390/ijms25063116

**Published:** 2024-03-08

**Authors:** Marina Raguž, Marko Tarle, Danko Müller, Čedna Tomasović-Lončarić, Hana Chudy, Tonko Marinović, Darko Chudy

**Affiliations:** 1 Department of Neurosurgery, Dubrava University Hospital, 10000 Zagreb, Croatia; marinaraguz@gmail.com (M.R.); tmarinovic@kbd.hr (T.M.); darko.chudy@gmail.com (D.C.); 2 School of Medicine, Catholic University of Croatia, 10000 Zagreb, Croatia; cedna.tomasovic-loncaric@unicath.hr; 3 Department of Maxillofacial Surgery, Dubrava University Hospital, 10000 Zagreb, Croatia; 4 School of Dental Medicine, University of Zagreb, 10000 Zagreb, Croatia; 5 Department of Pathology and Cytology, Dubrava University Hospital, 10000 Zagreb, Croatia; danko.mueller@yahoo.com; 6 School of Medicine, University of Zagreb, 10000 Zagreb, Croatia; 7 Department of Neurology, Dubrava University Hospital, 10000 Zagreb, Croatia; chudy.hana@gmail.com; 8 Medicine of Sports and Exercise Chair, Faculty of Kinesiology, University of Zagreb, 10000 Zagreb, Croatia

**Keywords:** glioma, ABCG2, CNS, tumor progression

## Abstract

Gliomas are notably challenging to treat due to their invasive nature and resistance to conventional therapies. The ABCG2 protein has attracted attention for its role in multidrug resistance, complicating treatment effectiveness. This study scrutinized the relationship between ABCG2 expression and glioma grade and the role of ABCG2 in the process of glioma progression, aiming to evaluate ABCG2 expression as a predictive factor of tumor progression and patient survival. Conducted at Dubrava University Hospital, Zagreb, Croatia, the study analyzed 152 glioma specimens from 2013 to 2022, assessing ABCG2 expression alongside standard clinical markers. A significant association was found between patients’ survival and the ABCG2 profile (*p* = 0.003, *r* = 0.24), separately for patients who underwent chemotherapy (*p* = 0.0004, *r* = 0.32) and radiotherapy (*p* = 0.003, *r* = 0.29). Furthermore, the ABCG2 profile was significantly associated with disease progression (*p* = 0.007, *r* = 0.23), tumor grade (*p* = 0.0002, *r* = 0.31), and Ki67 expression (*p* = 0.0004, *r* = 0.31). ABCG2-positive tumor cells only showed association with Ki67 expression (*p* = 0.002, *r* = 0.28). The ABCG2 profile was found to affect the overall patient survival (*p* = 0.02) and represent a moderate indicator of tumor progression (*p* = 0.01), unlike the percentage of ABCG2-positive tumor cells. ABCG2 may serve as a marker of angiogenesis and vascular abnormalities within tumors, predicting glioma progression and treatment response. Targeting ABCG2 could enhance chemoradiotherapy efficacy and improve patient outcomes, which highlights its value in assessing tumor aggressiveness and designing treatment strategies.

## 1. Introduction

Gliomas represent a diverse group of brain tumors that pose significant challenges in oncology, due to their complex biology and the intricacies of their treatment. The heterogeneity and complexity of these tumors result in poor prognosis and limited treatment options. Despite advances in neurosurgery, radiotherapy, and chemotherapy, the prognosis for patients with high-grade gliomas remains dismal, with median survival times that have seen only modest improvements over the past decades [1,2,3]. The complexity of glioma treatment is exacerbated by the brain’s unique environment, including the blood–brain barrier (BBB) that restricts the delivery of therapeutic agents [4]. Furthermore, the presence of glioma stem-like cells (GSCs) that contribute to resistance against conventional therapies has been extensively documented [5]. Cancer stem-like cells (CSCs) have gained significant attention for their potential to elucidate the persistence, recurrence, and treatment resistance observed in various cancers, including gliomas. CSCs can self-renew and differentiate into multiple cell types that constitute the tumor [5]. This attribute of CSCs is particularly interesting in the context of gliomas due to their role in promoting tumor growth, resistance, and relapse. The ATP-binding cassette sub-family G member 2 (ABCG2), also known as breast cancer resistance protein (BCRP), has emerged as a pivotal player in the multidrug resistance phenomenon observed in cancer treatment, including glioma treatment [6,7,8]. ABCG2 is a transmembrane protein acting as a drug efflux transporter that can limit the intracellular accumulation of chemotherapy agents, thereby diminishing their efficacy [9,10,11]. ABCG2 expression has been linked to the stem-like properties of glioma cells, including self-renewal and differentiation potential, which are characteristic of GSCs and most likely responsible for tumor initiation, recurrence, and resistance to conventional therapies [10]. In human glioma, ABCG2 is overexpressed in CSCs, underscoring the necessity for a deeper understanding of its biological and clinical implications. Studies have demonstrated that ABCG2 is not uniformly distributed across all tumor cells but is predominantly found in CSCs and in cells forming the BBB. This distribution pattern suggests a dual role for ABCG2 in protecting CSCs from chemotherapeutic agents and in modulating the permeability of the BBB to these agents. The implications of ABCG2 expression in gliomas extend beyond resistance mechanisms, as its presence has been correlated with tumor grade and patient prognosis [12,13]. High levels of ABCG2 expression are often associated with more aggressive tumor phenotypes and poorer outcomes, which highlights its potential as a prognostic marker for glioma patients [14]. Immunohistochemistry (IHC) has become an invaluable tool for the investigation of ABCG2 expression in glioma tissues, allowing for the precise localization and quantification of ABCG2 expression within the tumor microenvironment and offering insights into the biological behavior of gliomas and their response to therapy [11]. Studies utilizing IHC have demonstrated a correlation between high ABCG2 expression and increased tumor grade, particularly in CSCs and the tumor vasculature [15]. This expression pattern suggests a role for ABCG2 in the chemoresistant nature of these tumors and in maintaining the integrity of the BBB, even in the altered microenvironment of the tumor [11,12]. Given these complexities, the objective of this paper was to explore the expression of ABCG2 in human gliomas and its implications for treatment strategies and patient outcomes. This study aimed to scrutinize the relationship between ABCG2 expression and glioma grade, as well as the role of ABCG2 in the process of glioma grade progression, and try to detect the expression of ABCG2 as a potential predictive factor for tumor progression and patient survival.

## 2. Results

Between 1 January 2013 and 31 December 2022, a total of 152 consecutive patients (86 males, 56.6%, and 66 females, 43.4%) were enrolled in the study. The average age of the included patients was 47.23 ± 14.51 years. In our cohort, we included 78 grade II and 74 grade III glial tumors (Table 1).

In the analyzed group of patients, a statistically significant association was observed between the ABCG2 profile and tumor grade (*r* = 0.31, *p* = 0.0002), chemotherapy (*r* = 0.27, *p* = 0.002), Ki67 expression (*r* = 0.31, *p* = 0.0004), patients survival (*r* = −0.18, *p* = 0.02), progression (*r* = 0.23, *p* = 0.007), and radiotherapy (*r* = 0.17, *p* = 0.05). Regarding patients’ survival, the ABCG2 profile (*r* = −0.18, *p* = 0.02), chemotherapy (*r* = −0.25, *p* = 0.003), Ki67 expression (*r* = −0.19, *p* = 0.02), and radiotherapy (*r* = −0.22, *p* = 0.01) showed a significant association.

**Figure 1:**
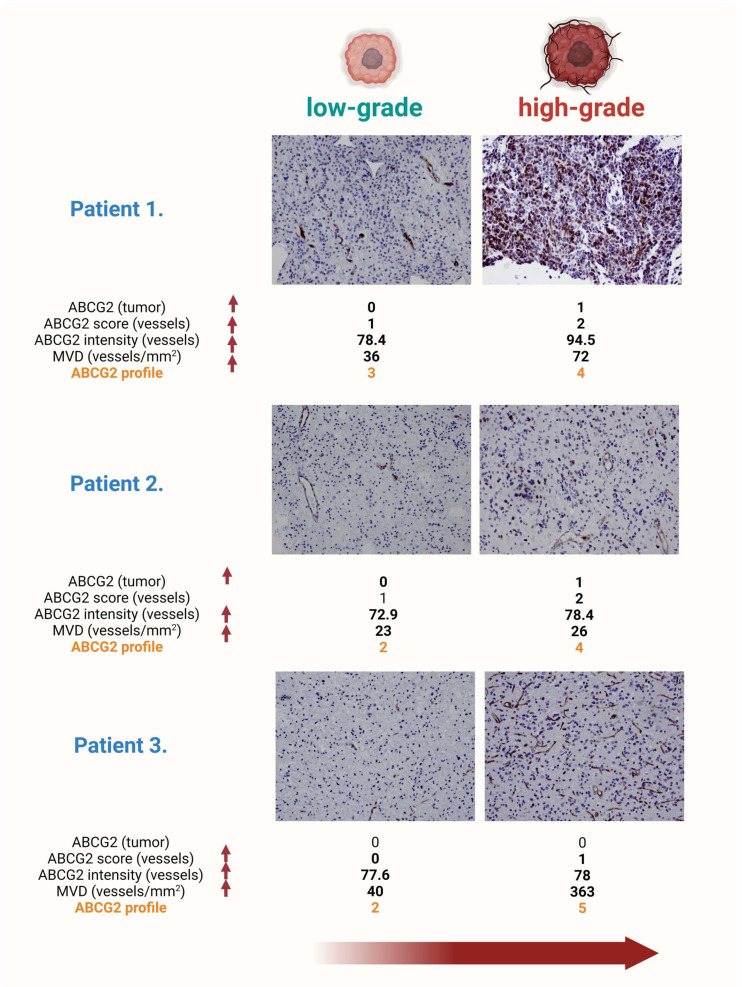
Progression of glioma grades and ABCG2 protein expression profiles in tumor tissue. The figure shows the histopathologic slides of three patients, illustrating the progression of gliomas from a low to a high grade. In the preparation of the first patient, progression is characterized by the onset of ABCG2 expression in the glioma tumor cells, architectural distortion of the blood vessels, increased ABCG2 expression in the endothelium, and an increase in microvascular density. Tumor progression in the second patient is characterized by the ABCG2 positivity of the tumor cells, a significant increase in vascular density, and increased staining intensity in the endothelium. In the third patient, a significant increase in the number of blood vessels is observed, accompanied by the appearance of branched capillaries and the enlargement of their lumen, indicating advanced angiogenic activity in high-grade gliomas. This triptych shows the dynamic molecular and structural changes during glioma progression and highlights the central role of ABCG2 expression as a marker and potential mediator of tumor vascularization and aggression. Magnification 200×.

Furthermore, the ABCG2 profile (*r* = 0.31, *p* = 0.0002), chemotherapy (*r* = 0.58, *p* < 0.0001), Ki67 expression (*r* = 0.69, *p* < 0.0001), tumor progression (*r* = 0.48, *p* < 0.0001), and radiotherapy (*r* = 0.50, *p* < 0.0001) showed a strong association with tumor grade (Table 2 and Figure 1).

ABCG2-positive tumor cells showed a strong association with other measured factors, such as the ABCG2 profile (*r* = 0.39, *p* < 0.0001) and Ki67 expression (*r* = 0.25, *p* = 0.004), as shown in Figure 1.

Interestingly, the ABCG2 profile showed no significant association with ATP-dependent helicase (ATRX) expression (*r* = 0.11, *p* = 0.25) nor with isocitrate dehydrogenase 1 (IDH-1) expression (*r* = −0.03, *p* = 0.72), while ABCG2-positive tumor cells showed no significant association with IDH-1 expression (*r* = 0.04, *p* = 0.65) but presented an association with ATRX expression (*r* = 0.19, *p* = 0.03).

Moreover, regression analysis showed a significant association between patients’ survival and the ABCG2 profile results, overall (R^2^ = 0.06, *p* = 0.003, *r* = 0.24) as well as in patients who underwent chemotherapy (R^2^ = 0.11, *p* = 0.0004, *r* = 0.32) and radiotherapy (R^2^ = 0.08, *p* = 0.003, *r* = 0.29) (Figure 1). Furthermore, the ABCG2 profile was significantly associated with progression to the higher grade of the tumor (R^2^ = 0.05, *p* = 0.007, *r* = 0.22), tumor grade (R^2^ = 0.07, *p* = 0.0008, *r* = 0.28), as well as Ki67 expression (R^2^ = 0.03, *p* = 0.04, *r* = 0.18).

**Figure 2:**
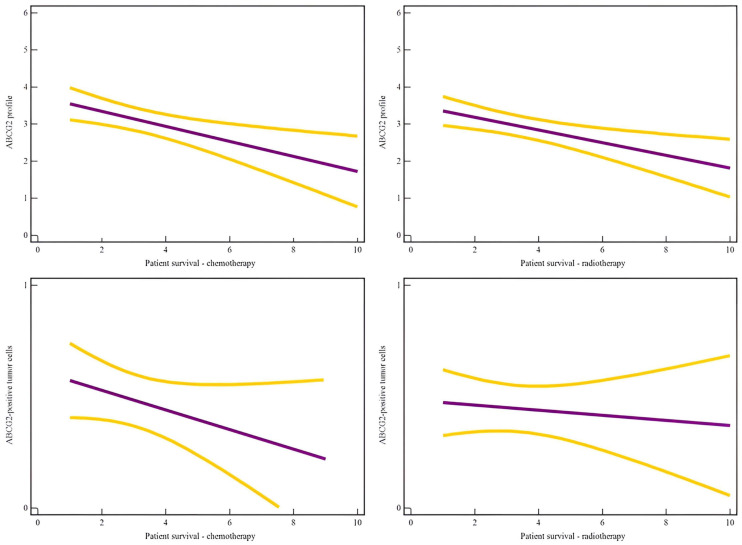
Regression analysis showed a significant association between patients’ survival and the ABCG2 profile in patients who underwent chemotherapy (R^2^ = 0.11, *p* = 0.0004, *r* = 0.32) and radiotherapy (R^2^ = 0.08, *p* = 0.003, *r* = 0.29), while no association was found between the percentage of ABCG2-positive tumor cells and patient survival in patients who underwent chemotherapy (R^2^ = 0.03, *p* = 0.16, *r* = 0.16) or radiotherapy (R^2^ = 0.002, *p* = 0.65, *r* = 0.05).

Regression analysis showed no association between the percentage of ABCG2-positive tumor cells and patient survival, overall (R^2^ = 0.003, *p* = 0.52, *r* = 0.06) as well as in patients who underwent chemotherapy (R^2^ = 0.03, *p* = 0.16, *r* = 0.16) or radiotherapy (R^2^ = 0.002, *p* = 0.65, *r* = 0.05) (Figure 2).

Furthermore, the percentage of ABCG2-positive tumor cells was not significantly associated with progression to the higher grade of the tumor (R^2^ = 0.005, *p* = 0.98, *r* = 0.0) nor with tumor grade (R^2^ = 0.006, *p* = 0.37, *r* = 0.08).

Still, the percentage of ABCG2-positive tumor cells was significantly associated with Ki67 expression (R^2^ = 0.07, *p* = 0.002, *r* = 0.28). MVD showed no significant association with overall survival (R^2^ = 0.001, *p* = 0.67, *r* = 0.04); still, it was significantly associated with progression to the higher grade of the tumor (R^2^ = 0.05, *p* = 0.007, *r* = 0.23), as well as with tumor grade (R^2^ = 0.05, *p* = 0.008, *r* = 0.23), respectively.

**Figure 3:**
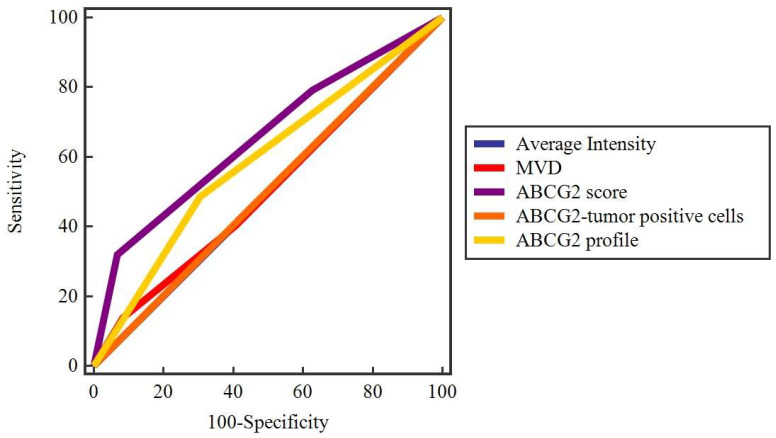
ROC curve analysis of the ABCG2 profile, ABCG2 score, microvascular density, average staining intensity, and percentage of ABCG2-positive tumor cells to evaluate their potential prognostic value for tumor progression. The ABCG2 profile (SE = 44.87, SP = 73.53, AUC = 0.59, Y = 0.18, *p* = 0.01) is a moderate indicator of tumor progression, as well as the ABCG2 score (SE = 31.94, SP = 93.22, AUC = 0.66, Y = 0.25, *p* = 0.0003).

**Figure 4:**
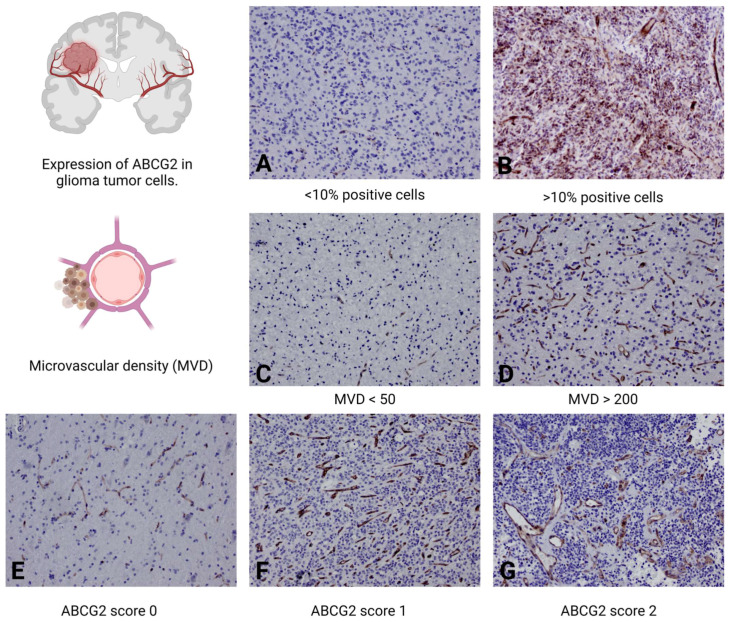
Immunohistochemical profiling of ABCG2 protein expression and microvascular density in glial tumors. Image of a tumor in which less than 10% of the tumor cells present cytoplasmic or membranous staining, indicating a lower severity of the disease (**A**) compared to a high-grade glioma that presents a significantly higher proportion of ABCG2-positive tumor cells, indicating increased protein expression (**B**). Low-grade glioma with microvascular density (MVD) characterized by less than 50 blood vessels per square millimeter of tumor tissue, indicating a less aggressive vascular profile (**C**) compared to that of a high-grade tumor, with an MVD of more than 200 blood vessels per square millimeter, indicating dense and potentially more aggressive angiogenic activity (**D**). In (**E**–**G**), tumors are classified according to the ABCG2 score from 0 to 2 to illustrate the extent of microvascular endothelial proliferation. A low-grade glioma with an ABCG2 score of 0 shows normal vascular morphology in the tumor tissue (**E**). In comparison, a high-grade glioma with a score of 1 shows a complex network of branched capillaries and an expanded endothelium (**F**). Finally, a high-grade glioma with an ABCG2 score of 2 is characterized by a pronounced accumulation of microvessels (**G**), indicating strong vascular proliferation. Magnification 200×.

Furthermore, to determine the value of the ABCG2 profile and the percentage of ABCG2-positive tumor cells in predicting potential complications, a ROC analysis was performed. The ABCG2 profile (SE = 44.87, SP = 73.53, AUC = 0.59, Y = 0.18, *p* = 0.01) was a moderate indicator of tumor progression, as well as the ABCG2 score (SE = 31.94, SP = 93.22, AUC = 0.66, Y = 0.25, *p* = 0.0003), while MVD (SE = 13.70, SP = 100.0, AUC = 0.55, Y = 0.14, *p* = 0.37), average staining intensity (SE = 100.0, SP = 1.67, AUC = 0.51, Y = 0.1, *p* = 0.32), and the percentage of ABCG2-positive tumor cells (SE = 56.16, SP = 44.07, AUC = 0.51, Y = 0.002, *p* = 0.98) appeared to be weak indicators of tumor progression (Figure 3 and Figure 4).

**Figure 5:**
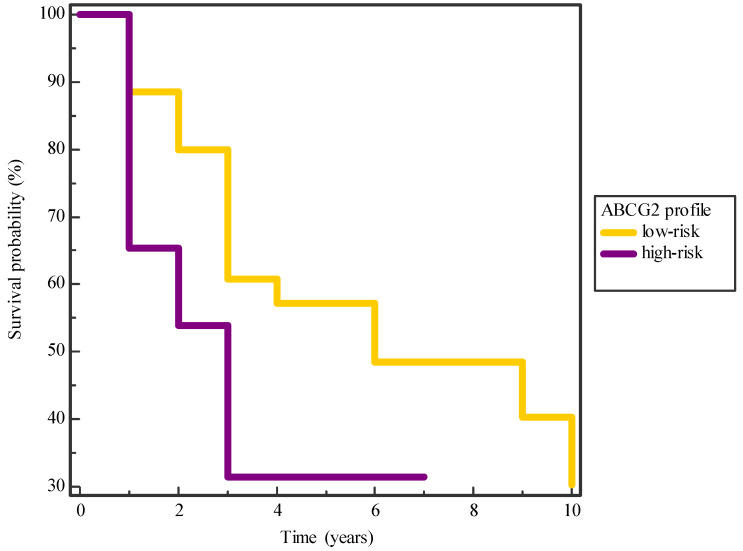
Kaplan–Meier survival curve considering the influence of the ABCG2 profile in glioma patients. The curve shows a trend of shorter survival for patients with higher ABCG2 profile values compared to those with lower values (*p* = 0.02).

Patients’ lifespan was followed from the time of diagnosis and/or surgery until the last follow-up or death. All patients were treated surgically. The median follow-up time was 3.71 ± 2.64 years, in a range of 1–10 years. During the follow-up period, 72/152 (51.8%) patients died, of which 17 died from another cause and were censored in the analysis of experience. In addition, tumor progression to a higher stage was observed in 78/152 (53.4%) patients during follow-up. The median time to disease progression was 2.53 ± 1.48 years. In the analysis of survival, the influence of the parameters on overall survival was first determined by the Kaplan–Meier method, and the difference between survival curves was determined by the log-rank test. The ABCG2 profile affected the overall patient survival (*p* = 0.02), although it did not affect patient survival in relation to chemotherapy (*p* = 0.06) and radiotherapy (*p* = 0.07); also the percentage of ABCG2-positive tumor cells (*p* = 0.87, *p* = 0.77, *p* = 0.61) did not affect patient survival, as shown in Figure 5. A difference in survival between patients was found in relation to IDH-1 expression (*p* = 0.0001) and tumor progression (*p* < 0.0001), while the previously described parameters did not affect the overall patient survival.

## 3. Discussion

In this study, we aimed to explore the expression of ABCG2 and its association with glioma grade and tumor progression, as well as to evaluate the expression of ABCG2 as a potential predictive factor for tumor progression and patient survival.

Most previous studies explored ABCG2 expression in grade IV glioma, as well as differences in expression in low- and high-grade tumors in animal models [16,17,18,19,20]. Animal studies, particularly those utilizing rodent models of glioma, have been instrumental in elucidating the role of ABCG2 in the BBB and the treatment of gliomas. Studies using glioma xenografts in mice showed that ABCG2 contributes to the formation and maintenance of the BBB, thereby influencing drug delivery and therapeutic outcomes in patients with glioma. These findings are pivotal, considering the critical role of the BBB in protecting the brain from toxic substances, including chemotherapeutic agents [21]. Furthermore, the inhibition or knockdown of ABCG2 in rodent models can enhance the accumulation of chemotherapeutic drugs in the brain, suggesting a potential strategy to improve drug delivery and efficacy in glioma treatment [22]. In addition, animal studies have facilitated the understanding of the BBB role in drug resistance, with ABCG2 being a critical component in the function of the BBB, influencing drug efflux from the brain [6,7]. These models have provided valuable insights into the regulation of ABCG2 expression under pathological conditions, such as in the presence of a tumor, revealing the dynamic nature of the BBB and the challenges it poses in the context of drug delivery. Research in both human samples and animal models also revealed that ABCG2 interacts with other molecular pathways involved in drug resistance and tumor progression, such as the phosphoinositide 3-kinase/protein kinase B/mammalian target of rapamycin (PI3K/Akt/mTOR) pathway, a crucial signaling pathway implicated in cell growth, survival, and drug resistance in glioma [23]. These interactions highlight the complexity of ABCG2 role in glioma and underscore the need for comprehensive strategies targeting multiple pathways to overcome drug resistance. IHC analysis in these models showed that the modulation of ABCG2 expression affects drug distribution within the tumor and the surrounding brain tissue, impacting the efficacy of chemotherapeutic agents [22]. In general, IHC studies have been instrumental in localizing and quantifying ABCG2 expression within tumor tissues, revealing a heterogeneous distribution of ABCG2 within glioma tissues [21]. Notably, ABCG2 staining is predominantly observed in CSCs and perivascular niches, suggesting a role in maintaining stemness and the BBB [8,11]. This specific localization pattern correlates with the CSC theory of cancer, supporting the role of ABCG2 in maintaining the stem cell phenotype and its contribution to the resilience of these tumors to chemotherapy [12]. CSCs, identified by their ABCG2 expression, have been implicated in tumor recurrence and poor prognosis, which underlines their clinical significance as a biomarker [8,24]. ABCG2 is expressed not only in tumor cells but also on the luminal surface of brain capillary endothelial cells within and surrounding the tumor. IHC studies revealed that ABCG2 is a component of the BBB and the blood–tumor barrier (BTB), where it functions as an efflux transporter. This efflux transporter plays a pivotal role in maintaining the homeostasis of the brain’s microenvironment by actively pumping out xenobiotics and endogenous toxins, thus protecting the neural tissue from potential damage [6,7]. Additionally, the upregulation of ABCG2 in the vasculature was linked to the efflux of drugs from the brain, contributing to the chemoresistant nature of gliomas [16,17,18]. Thus, the expression of ABCG2 in the vasculature is of particular interest, as it influences the permeability to chemotherapeutic drugs, affecting their efficacy [25,26]. The intensity and pattern of ABCG2 staining have been correlated with the grade of malignancy in gliomas. Higher levels of ABCG2 expression are generally associated with higher grade tumors, indicating a more aggressive and treatment-resistant phenotype, suggesting ABCG2 potential as a prognostic marker [16,17,18,19,20,23,26]. This also highlights the importance of considering the ABCG2 status in the stratification of patients for tailored therapeutic approaches. Studies showed that patients with tumors exhibiting high ABCG2 expression have a poorer prognosis, suggesting that ABCG2 could be a valuable target for therapeutic intervention [12]. In our cohort, a significant association was found between patients’ survival and the ABCG2 profile results, overall as well as in patients who underwent chemotherapy and radiotherapy. Patients with glioma tumors with higher ABCG2 expression exhibited poorer survival rates following chemotherapy or radiotherapy treatment [27]. As mentioned previously, the expression of ABCG2 has been linked to the effectiveness of chemotherapy and radiotherapy, also in patients with brain tumors [6,7,28]. In the context of chemotherapy, the overexpression of ABCG2 can lead to decreased drug accumulation, rendering chemotherapy less effective, due to the ability of ABCG2 to promote the efflux of a variety of chemotherapeutic agents, including topotecan, methotrexate, and doxorubicin, among others [14]. As a result, tumors with high levels of ABCG2 expression may show resistance to these drugs, making it challenging to achieve therapeutic efficacy. On the other hand, the relationship between ABCG2 expression and radiotherapy effectiveness is less direct but still significant. While ABCG2 primary function is related to drug efflux, its expression can also be an indicator of a CSC population within the tumor. CSCs are believed to be more resistant to conventional therapies, including radiotherapy, due to their enhanced DNA repair capabilities, quiescent state, and other intrinsic survival mechanisms. Therefore, high ABCG2 expression might correlate with a higher proportion of CSCs in the tumor, potentially leading to increased resistance to radiotherapy [8,11,29,30].

The localization of ABCG2 within the tumor vasculature significantly complicates the landscape of glioma treatment. The BBB, a selective barrier designed to protect the brain from potentially harmful substances, also poses a major obstacle to the delivery of drugs to brain tumors. The expression of ABCG2 at the BBB contributes to this challenge by actively promoting the transport of many chemotherapy agents back into the bloodstream, thereby reducing their therapeutic concentrations within the brain. This efflux activity of ABCG2 at the BBB underscores the need for innovative strategies that can either bypass or inhibit ABCG2 function to enhance the delivery of chemotherapeutic drugs to gliomas [6,7,11,27,28,31]. The ABCG2 expression pattern provides valuable insights into endothelial proliferation and changes in vascular architecture within tumors. Thus, the ABCG2 profile, which encompasses ABCG2 score, microvascular density, and staining intensity in the tumor’s blood vessels, plays a crucial role in predicting tumor progression and survival in patients with glioma tumors, as well as tumor resistance to chemoradiotherapy. This profile helps in understanding the tumor microenvironment’s dynamics, especially how it influences drug delivery and efficacy. By assessing ABCG2 expression in the tumor vasculature, clinicians can better gauge the aggressiveness of gliomas and tailor treatment strategies accordingly. Therefore, the ABCG2 profile and expression levels serve as significant markers for both the biological behavior of gliomas and their response to therapeutic interventions.

ABCG2 has been identified not only in cancer cells but also in various types of normal tissues, including the endothelial cells lining blood vessels [8]. The presence of ABCG2 in both cancer cells and the endothelium of blood vessels suggests a multifaceted role in physiological processes, such as the protection against xenobiotics, and in the pharmacokinetics of drugs. In cancer cells, ABCG2 plays a critical role in promoting the multidrug resistance phenotype, as it actively enhances the efflux of chemotherapeutic agents, leading to decreased intracellular drug accumulation and a reduced efficacy of cancer treatments [32,33]. Its expression is often upregulated in response to chemotherapy, making it a target for overcoming drug resistance in cancer therapy. In the endothelial cells of blood vessels, ABCG2 contributes to the BBB and the BTB, among others, by restricting the entry of toxic substances and xenobiotics into protected tissue spaces. This protective function extends to the efflux of drugs from the bloodstream into the extracellular space, influencing the distribution and elimination of many pharmacological agents [6,7,34,35]. In the context of the BBB, ABCG2 helps to maintain brain homeostasis by preventing neurotoxic compounds from entering the central nervous system. The connection between ABCG2-expressing cancer cells and blood vessels is significant for several reasons. The expression of ABCG2 in the endothelial cells of blood vessels can limit the delivery of chemotherapeutic drugs to the tumor microenvironment, affecting treatment efficacy. ABCG2 is considered a marker of CSCs, which are thought to be responsible for tumor initiation, metastasis, and resistance to therapy. CSCs residing in niches close to blood vessels may exploit the local ABCG2 expression to protect themselves from therapeutic agents. Although the direct role of ABCG2 in tumor angiogenesis is less clear, the protein’s presence in endothelial cells suggests it might influence the tumor microenvironment and the development of new blood vessels, which are essential for tumor growth and metastasis. Understanding the role of ABCG2 in both cancer cells and blood vessels may help unravel the complexity of drug resistance and highlight the need for strategies that can bypass or inhibit ABCG2 function [29].

The prognostic value of ABCG2 expression in gliomas has been increasingly recognized. Studies demonstrated that higher levels of ABCG2 correlate not only with a more aggressive tumor phenotype but also with poorer patient survival rates. Our study showed significant associations between ABCG2 expression and tumor grade and progression as well as patient survival, while the percentage of ABCG2-positive tumor cells was not significantly associated with tumor progression or tumor grade. Both ABCG2 expression and the percentage of ABCG2-positive tumor cells were significantly associated with Ki67 expression. The Ki67 marker is a well-established indicator of cellular proliferation, serving as a critical factor in assessing the aggressiveness of glioma brain tumors [36]. Its expression levels directly correlate with the growth rate of tumor cells, providing valuable prognostic information regarding tumor behavior and potential progression. High Ki67 labeling indices are often associated with aggressive gliomas, indicating a poore prognosis and a high likelihood of rapid disease progression [1]. Consequently, Ki67 expression is utilized in neuropathology to stratify glioma patients into different risk categories, guiding treatment decisions and helping to predict the response to therapy. In our cohort, we presented a significant association between Ki67 expression and ABCG2 profile in human gliomas. Similar results were presented for invasive breast ductal carcinoma, suggesting a regulatory mechanism impacting tumor aggressiveness and multidrug resistance. High Ki67 levels, indicative of cell proliferation, along with high expression of ABCG2, a known multidrug resistance protein, point towards a complex interaction influenced by hypoxia conditions within tumors, potentially affecting chemotherapy outcomes and patient prognosis [37]. Interestingly, the ABCG2 score showed no significant association with ATRX or IDH-1 expression, while the percentage of ABCG2-positive tumor cells showed no significant association with IDH-1 expression but presented an association with ATRX expression. The association between ATRX and ABCG2 levels in brain tumors, particularly in gliomas, highlights the complex interplay of genetic alterations and drug resistance mechanisms that influence tumor behavior and therapeutic outcomes. ATRX is a chromatin remodeler that plays a crucial role in telomere maintenance, gene expression regulation, and DNA repair. Mutations in the ATRX gene are frequently observed in certain types of gliomas, where they are associated with the alternative lengthening of telomeres phenotype, genomic instability, and altered epigenetic landscapes. ATRX loss is often correlated with poor prognosis, increased tumor aggressiveness, and a specific molecular subtype of glioma. There are several potential mechanisms underlying the implications of the interplay between ATRX mutation status and ABCG2 expression in gliomas. ATRX mutations may contribute to a tumor microenvironment that favors the expression of drug resistance genes, including ABCG2. This could be mediated through alterations in chromatin structure and gene expression patterns in tumor cells. Additionally, the loss of ATRX function could lead to changes in the tumor microenvironment that indirectly upregulate ABCG2 expression, either through hypoxic responses or by affecting the cellular composition of the tumor, such as the presence of cancer stem cells, which are known to express high levels of ABCG2. Understanding the relationship between ATRX status and ABCG2 expression could provide insights into the prognosis of glioma patients and inform treatment strategies. For instance, tumors with ATRX mutations and high ABCG2 expression might be more resistant to certain chemotherapies, suggesting the need for alternative therapeutic approaches or the use of ABCG2 inhibitors to enhance drug efficacy [38,39,40]. It is important to note that while the potential correlation between ATRX mutations and ABCG2 expression in brain tumors is based on their roles in genomic stability, drug resistance, and tumor biology, direct evidence and detailed mechanisms of their interaction remain to be fully elucidated through further research.

Although a significant association was presented between patient survival and the ABCG2 profile, overall and in patients who underwent chemotherapy and radiotherapy, the percentage of ABCG2-positive tumor cells showed no association with patient survival. This association underscores the potential of ABCG2 as a prognostic biomarker, enabling more personalized treatment approaches based on the molecular characteristics of the tumor [41,42]. Additionally, both ABCG2 profile and score were shown to be moderately associated with tumor progression, while the percentage of ABCG2-positive tumor cells was shown to be a weak indicator of tumor progression. Regarding patient survival, the ABCG2 score affected patient survival overall but not in relation to chemotherapy and radiotherapy, and the level of ABCG2 tumor cell expression did not affect patient survival.

Moreover, the exploration of ABCG2 inhibitors, both in vitro and in vivo, has provided promising insights into overcoming drug resistance [43]. Small-molecule inhibitors and monoclonal antibodies against ABCG2 were shown to effectively sensitize resistant cancer cells to chemotherapy [44]. The exploration of ABCG2 inhibitors used in conjunction with traditional chemotherapy represents a promising approach to surmount drug resistance in gliomas. The advancement of novel drug delivery systems capable of circumventing ABCG2-mediated drug efflux at the BBB holds the potential to radically transform the treatment paradigm for brain tumors [45,46]. Furthermore, recent advancements in nanotechnology offer novel avenues for circumventing ABCG2-mediated drug resistance. Nanoparticle-based drug delivery systems have been engineered to bypass ABCG2-promoted drug efflux, ensuring higher intracellular concentrations of chemotherapeutic agents. These innovative strategies represent a significant leap forward in the quest to improve the therapeutic outcomes for glioma patients [47]. Additionally, the use of RNA interference technology to knock down ABCG2 expression has yielded promising results in preclinical models, further supporting the therapeutic potential of targeting ABCG2 in glioma treatment [48]. Extracellular vesicles, particles naturally released from cells passing the BBB in a bi-directional manner between the bloodstream and the brain parenchyma, could also be used as possible carriers for drugs targeted to the brain [49]. Despite these advancements, several challenges remain in translating these findings into clinical practice. The heterogeneity of gliomas and the complexity of the BBB pose significant obstacles to the effective delivery of ABCG2 inhibitors. Moreover, the safety and specificity of these inhibitors need to be thoroughly assessed to avoid adverse effects on normal tissues expressing ABCG2 [50].

ABCG2 has proven to be an excellent marker of angiogenesis, accurately indicating disturbances in the architecture of blood vessels within tumors. It serves as a reliable indicator of endothelial proliferation, and determining the proposed profile can aid in predicting the progression of glial tumors as well as their response to chemoradiotherapy. This marker’s ability to reflect changes in vascular structure and function makes it invaluable for assessing tumor aggressiveness and potential treatment strategies. Furthermore, the ABCG2 profile’s predictive value for therapy outcomes highlights its importance in personalized medicine approaches for glioma patients. By integrating ABCG2 profiling into clinical practice, oncologists can better tailor treatment plans, potentially improving survival rates and quality of life for those affected by not only gliomas, but also all other human cancers [51].

The limitations of the study include its retrospective design, which inherently carries the risk of selection and recall bias. The fact that some patients were lost to follow-up or continued their care at other facilities introduced an attrition bias, complicating the assessment of long-term outcomes such as survival rates and disease progression. The use of experimental antibodies for ABCG2 detection may not be as reliable or validated as that of established antibodies, raising concerns about the specificity and reproducibility of the results. Lastly, these combined factors limit the generalizability of the study’s conclusions to broader patient populations and different clinical settings, suggesting a cautious interpretation of the findings and the need for further validation in prospective studies.

## 4. Materials and Methods

### 4.1. Patients

In this retrospective study, we analyzed data from the electronic medical records and paraffin-embedded archival specimens from biopsies and open surgeries of gliomas grade I, II, and III at the Department of Neurosurgery and the Department of Pathology and Cytology, Dubrava University Hospital, Zagreb, Croatia, between 1 January 2013 and 31 December 2022. We included 152 consecutive patients admitted and treated surgically. To confirm the diagnosis and to determine the adequacy of the quality and quantity of the pathohistological material, two pathologists from Dubrava University Hospital examined the subjects’ specimens again, separately. To be included in this study, patients had to meet the following criteria: (a) clinically and patohistologically proven glioma sampled by stereotactic biopsy or open surgery, (b) available pathohistological material for immunohistochemical analysis, (c) clinically and patohistologically relevant data from medical history, the hospital information system, the clinical oncology database, and the cancer registry of the Croatian Institute of Public Health. The exclusion criteria were as follows: (a) absent pathohistological material mandatory for quantitative analysis, (b) other types of tumor processes, (c) incomplete medical documentation. This study was carried out following the recommendations of the ethics board of the Dubrava University Hospital. Written informed consent was obtained from all patients, following the Declaration of Helsinki. The protocol was approved by the Institutional Review Board of the Dubrava University Hospital, Zagreb, Croatia (2022/1512-04).

### 4.2. Pathohistological Samples

Paraffin-embedded archival specimens from biopsies of gliomas sampled from stereotactic biopsies or open surgery were used for this study. To confirm the diagnosis and to determine the adequacy of the quality and quantity of the pathohistological material, two neuropathologists from the Department of Pathology and Cytology of Dubrava University Hospital examined the subjects’ specimens again, separately. The specimens were fixed in 10% buffered formalin (Kemika, Zagreb, Croatia), embedded in paraffin, cut into 3 to 4 µm thick sections, deparaffinized, and stained with hemalaun–eosin (HE).

### 4.3. Immunohistochemical Staining

In this study, 2–3 µm thick sections were prepared from the paraffin blocks and then dewaxed in a thermostat. After deparaffinization, predigestion was performed in a thermobath (PT-link, DAKO, Glostrup, Denmark), followed by treatment with the EnVision target Retrieval solution, High pH (DAKO, Glostrup, Denmark), i.e., predigestion with exposure of epitopes by heat in a microwave oven in pH 6 buffer to determine the expression of the ABCG2 protein. Immunohistochemical staining was performed using an automated immunohistochemical system (DAKO autostainer, DAKO, Glostrup, Denmark). For immunohistochemical staining, a ready-to-use ABCG2 antibody, clone B-1, at a dilution of 1:25 was used in a 90 min incubation. Immunohistochemical staining expression was detected by an indirect method using the EnVision detection kit (DAKO, Glostrup, Denmark). Subsequently, the preparations were contrasted with hemalaun (1 min) and placed in an ascending series of alcohol (70–100%) and then in xylene and finally sealed with a glass coverslip. Paraffin-embedded breast tissue served as a positive control for ABCG2, according to the recommendations of the manufacturer of the antibodies tested.

The Ki-67 marker’s immunohistochemical evaluation was conducted using the Ventana BenchMark Ultra system (Roche Diagnostics, Tucson, AZ, USA) for predigestion, following deparaffinization and cell conditioning. An automated system facilitated the staining process, employing the optiView Universal DAB Detection kit (Ventana Medical Systems, Tucson, AZ, USA) for the marker’s visualization. A mouse monoclonal anti-Ki67 antibody (clone MIB-1, DAKO, Glostrup, Denmark), diluted to 1:75 and incubated with the samples for 16 min at 37 °C, was utilized. Visualization involved hydrogen peroxide and the DAB chromogen, creating a brown deposit observable via light microscopy, with hemalaun contrasting, and a dehydration sequence culminating in xylene treatment and coverslipping. Tonsil tissue served as a positive control.

### 4.4. Evaluation of the Immunohistochemical Staining

The immunohistochemical analysis of the expression of IDH1, ATRX, Ki67, and GFAP in glioma tumors was meticulously carried out in line with globally recognized protocols, ensuring a standardized approach to evaluate these critical markers [52].

The immunoreactivity of the ABCG2 protein was evaluated in glioma tumor cells and in the endothelium of blood vessels within the tumors (ABCG2 score, MVD, average staining intensity).

#### 4.4.1. ABCG2 Expression in Tumor Cells (0–1)

To evaluate the immunoreactivity of the ABCG2 protein on the membrane or in the cytoplasm of tumor cells, a scoring system described by Jin et al. was employed [19]. The percentage of positive cells was recorded in hot spots for each specimen at 200× magnification using a light microscope. Gliomas with no positive tumor cells or with less than 10% positive cells were scored as 0, whereas tumors with more than 10% positive cells were scored as 1.

#### 4.4.2. ABCG2 Expression in the Endothelium (ABCG2 Score 0–2)

For the semiquantitative analysis of ABCG2 in the endothelium of brain tumors, microvascular proliferation patterns were assessed based on a scoring range (0–2) similar to that previously established for CD34 staining [53,54]. The scores were assigned as follows: 0 for physiological microvessel appearance, 1 for branching capillaries with expanded endothelia and slightly increased lumina, and 2 for clusters of microvessels.

#### 4.4.3. Microvascular Density (MVD)

In this study, microvascular density (MVD) in glioma brain tumors was quantified through histological examination. Tumor sections were analyzed under a light microscope at a magnification of 200×, focusing on the areas of highest neovascularization, known as ‘hot spots’. Within these regions, all discernible blood vessels were counted within a field of view encompassing 1 mm^2^. This approach allowed for the precise assessment of MVD, providing insights into the vascular characteristics of the gliomas under investigation.

#### 4.4.4. Average Staining Intensity of ABCG2 in the Endothelium

To determine the average staining intensity of ABCG2 in endothelial cells, excluding tumor cells, the ImageJ software (Version 1.54h; NIH, Bethesda, MD, USA) was utilized. Digital images of tissue sections were processed, and specific areas containing only endothelial cells were selected using the Region of Interest (ROI) tool (ImageJ software—Version 1.54h; NIH, Bethesda, MD, USA). This approach facilitated the quantitative analysis of ABCG2 expression, focusing exclusively on the targeted cell population.

All analyses were conducted in collaboration with neuropathologists (D.M. and Č.T.-L.). In cases of discrepancy, discussions were held until a consensus was reached.

### 4.5. ABCG2 Profile

To refine our approach in assessing glioma tumors, we devised an ABCG2 profile scoring system (0–6 points), encompassing several parameters indicative of the vascular pattern in gliomal tumors, i.e., microvascular density (MVD) + average staining intensity of ABCG2 in the endothelium + ABCG2 score.

The scoring mechanism for the ABCG2 profile was designed as follows: MVD was evaluated based on the density of blood vessels within the tumor—assigning 0 points for less than 100 vessels/mm^2^, 1 point for a density of 100–200 vessels/mm^2^, and 2 points for densities exceeding 200 vessels/mm^2^. In a similar vein, the intensity of ABCG2 expression in blood vessels was scored with 0 points for expression of less than 50%, 1 point for expression between 50–75%, and 2 points for expression exceeding 75%. The ABCG2 score was determined by summing the points from these categories, as previously outlined, ranging from 0 to 2.

Tumors achieving a cumulative score of less than 3 were deemed low-risk, indicative of a potentially less aggressive phenotype. On the other hand, tumors with a total score of 3 or higher were classified as high-risk, suggesting a likelihood of more aggressive tumor behavior.

### 4.6. Statistical Analysis

Statistical processing of the data was performed with the statistical computer program MedCalc, version 12.5.0 (MedCalc Software, Ostend, Belgium; https://www.medcalc.org, accessed on 10 February 2024), and the results are presented in tables and graphs. The values of continuous variables are presented as mean ± standard deviation. The analysis of the distribution of the measured variables (Kolmogorov–Smirnov test) determined the difference in the distribution of each variable; the normality of the distribution varied from parameter to parameter. Associations (correlations) between individual parameters were examined using the Pearson test or Spearman test and the regression model, depending on the normality of the data distribution. The relationship between the expression of ABCG2 and the overall survival of the subjects was assessed by the Kaplan–Meier method, and the log-rank test determined the difference between the survival curves. The potential prognostic value of the analyzed biomarkers was determined with ROC (Receiver Operating Characteristic) analysis. The test results were considered significant when *p* ≤ 0.05.

## 5. Conclusions

Our research clarified the expression of ABCG2 in human gliomas and suggests possible implications for treatment strategies and patient outcomes. Furthermore, our results indicate a correlation between ABCG2 expression and tumor grade, tumor progression, and patients’ survival, suggesting ABCG2 expression as a potential predictive factor for tumor progression and patient survival and illuminating the potential of targeting ABCG2 as a therapeutic strategy to eliminate the resilient cell population expressing ABCG2 and improve therapeutic outcomes. It is expected that these and similar results will be important for clinicians to best choose the therapeutic modality for an individual patient, improving disease control with the aim of better survival, reducing the harmfulness of therapy, and preserving the patient’s quality of life.

## Figures and Tables

**Table 1:** Demographic and clinical data of the patients included in the study.

	Male	Female	Total
No of patients	86 (56.6%)	66 (43.4%)	152 (100%)
Age (years)	46.7 ± 14.8	46.9 ± 15.2	47.23 ± 14.51
Grade II/Grade III	44/42	34/32	78/74

**Table 2:** Associations of the ABCG2 profile and the percentage of ABCG2-positive tumor cells with other molecular biomarkers, clinical data, and analyzed features of ABCG2 expression.

		ABCG2 Profile	Grade	Chemoth	Ki67	Survival	Progression	Radioth	Positive Tumor	ATRX	IDH-1
ABCG2 profile	*r* *p*		0.31 0.0002	0.27 0.002	0.31 0.0004	−0.187 0.02	0.23 0.007	0.17 0.05	0.39 <0.0001	0.11 0.25	−0.03 0.72
Grade	*r* *p*	0.31 0.0002		0.58 <0.0001	0.69 <0.0001	−0.15 0.07	0.48 <0.0001	0.50 <0.0001	0.075 0.39	0.04 0.67	0.11 0.19
Chemoth	*r* *p*	0.27 0.002	0.58 <0.0001		0.49 <0.0001	−0.25 0.003	0.56 <0.0001	0.63 <0.0001	0.11 0.22	0.02 0.83	−0.06 0.50
Ki67	*r* *p*	0.31 0.0004	0.69 <0.0001	0.49 <0.0001		−0.19 0.02	0.39 <0.0001	0.39 <0.0001	0.25 0.004	0.23 0.01	−0.03 0.74
Survival	*r* *p*	−0.18 0.02	−0.15 0.07	−0.25 0.003	−0.19 0.02		−0.06 0.45	−0.22 0.01	−0.05 0.55	−0.16 0.06	0.23 0.009
Progression	*r* *p*	0.23 0.007	0.48 <0.0001	0.56 <0.0001	0.39 <0.0001	−0.06 0.45		0.44 <0.0001	−0.002 0.98	−0.19 0.02	0.11 0.25
Radioth	*r* *p*	0.17 0.05	0.50 <0.0001	0.63 <0.0001	0.39 <0.000	−0.22 0.01	0.44 <0.0001		0.04 0.68	0.07 0.41	−0.02 0.80
Positive tumor	*r* *p*	0.39 <0.0001	0.07 0.39	0.11 0.22	0.25 0.004	−0.05 0.56	−0.002 0.98	0.04 0.68		0.19 0.03	0.04 0.65
ATRX	*r* *p*	0.11 0.25	0.04 0.67	0.02 0.83	0.23 0.01	−0.16 0.06	−0.19 0.02	0.08 0.41	0.19 0.03		−0.02 0.82
IDH-1	*r* *p*	−0.03 0.72	0.11 0.19	−0.06 0.50	−0.03 0.74	0.23 0.009	0.11 0.25	−0.02 0.81	0.04 0.65	−0.02 0.82	

## Data Availability

All data generated or analyzed during this study are included in this published article.
